# Fracture Surface Morphology and Impact Strength of Cellulose/PLA Composites

**DOI:** 10.3390/ma10060624

**Published:** 2017-06-07

**Authors:** Honghong Gao, Tao Qiang

**Affiliations:** 1 School of Mechatronic Engineering, Xi’an Technological University, Xi’an 710021, China; 2 School of Materials Science & Chemical Engineering, Xi’an Technological University, Xi’an 710021, China; qiangtao2005@163.com

**Keywords:** cellulose, polylactide, extrusion blending, injection molding, impact strength, fractal analysis

## Abstract

Polylactide (PLA)-based composite materials reinforced with ball-milled celluloses were manufactured by extrusion blending followed by injection molding. Their surface morphology from impact fracture were imaged with scanning electron microscopy (SEM) and investigated by calculating their fractal dimensions. Then, linear regression was used to explore the relationship between fractal dimension and impact strength of the resultant cellulose/PLA composite materials. The results show that filling the ball-milled celluloses into PLA can improve the impact toughness of PLA by a minimum of 38%. It was demonstrated that the fracture pattern of the cellulose/PLA composite materials is different from that of pristine PLA. For the resultant composite materials, the fractal dimension of the impact fractured surfaces increased with increasing filling content and decreasing particle size of the ball-milled cellulose particles. There were highly positive correlations between fractal dimension of the fractured surfaces and impact strength of the cellulose/PLA composites. However, the linearity between fractal dimension and impact strength were different for the different methods, due to their different R-squared values. The approach presented in this work will help to understand the structure–property relationships of composite materials from a new perspective.

## 1. Introduction

Polylactide (PLA) has been regarded as the best substitute for various petroleum-based polymers in the framework of environmentally-friendly processes and products for the past decades, due to its renewability, degradability, biocompatibility, and good thermomechanical properties [1,2]. PLA is a semi-crystalline thermoplastic which exhibits high strength and stiffness comparable to those of polystyrene (PS). Furthermore, it can degrade to nontoxic compounds in landfills [3]. However, several disadvantages impede many potential applications. The most major problem is its inherent brittleness in the pristine state [4]. Several approaches, including plasticization, copolymerization, and melt blending with flexible polymers, were developed to toughen PLA in the past years [5]. The second issue with PLA is its relatively high cost. Therefore, low-cost natural fibers such as plant and animal fibers have been used to modify PLA to fabricate cost-efficient composite materials with improved toughness [6,7]. Among them, cellulose is one of the most abundant renewable natural polymers with enormous advantages, such as low density, worldwide availability, biodegradability, non-toxicity, and good mechanical properties [8,9,10]. Cellulose is a fibrous and water-insoluble biomaterial in nature. The widespread applications of cellulose reinforcements in environmentally-benign polymers exhibited considerable promise for manufacturing 100% bio-based, renewable, and degradable composites with desired physical properties, such as acceptable specific strength, low density, high toughness, and improved thermal properties [11,12]. However, raw cellulose fibers are hard to directly disperse into polymer matrices uniformly via extrusion blending and injection molding, due to their large aspect ratio and entangled structures. So, ball-milling is used to access the desired fillers at first in a facile and solvent-free route [13]. Then, extrusion blending followed by injection molding can be used to fabricate PLA-based composites reinforced with ball-milled cellulose particles via conventional melting processing.

Fracture surfaces that usually are rough and irregular play important roles in several areas, such as the design, fabrication, evaluation, and application of materials. To some extent, fracture surfaces reflect the impact or tensile resistance of the resulting materials [14]. Many methods can be used to analyze and describe the fractured surface. Among them, imaging methods such as scanning electron microscopy (SEM) and atomic force microscopy (AFM) are widely used for surface visualization and characterization.

Since the fractal character (i.e., self-similarity) of fracture surfaces of metals was reported by Mandelbrot et al. in 1984 [14], fractal dimension, D*_f_*, has been used as a measure of the self-affine nature of materials, such as thin film [15] and nano-materials [16]. In reference [15], D*_f_* is used to determine the irregularity of the thin film surfaces. On this basis, a theoretical equation was developed to demonstrate the relationship between D*_f_* and surface energy [15]. In nature, self-affinity is a generalization of self-similarity, which is the basic property of most of the deterministic fractals. According to another report, strong linear correlation is confirmed between fractal dimensions of the fracture surface and V-notched impact strength of poly-l-lactide (PLLA) composites filled with nano-sized calcium carbonate [17]. In addition, linear correlation was confirmed between their fracture surface topographies and impact strength of the PLA-based wood plastic composites (WPC) toughened with polyhydroxyalkanoates (PHAs) [18], linear low density polyethylene (LLDPE) [19], and styrene–butadiene–styrene (SBS) block copolymer [20] via fracture surface analysis.

The Gwyddion is a modular program for scanning probe microscopy (SPM) data analysis. This free and open-source software can be used for any other height field analysis or image analysis, although it was primarily supposed to analyze height fields obtained via SPM technique [15,21,22]. It is well known that SEM is one of the excellent tools for intuitive visualization and qualitative description of surface morphology. In recent years, surface structure and roughness obtained by SEM has been used as an indirect image-based method for materials [23,24]. For example, the fractal structures of SiO_2_ nanoparticles on silicon substrates [16] and graphene particles [25] have been verified with the Gwyddion software using their individual SEM images. Additionally, the Gwyddion was used to investigate the correlations between their fracture surface morphologies and impact strength of the PLA-based wood plastic composites (WPC) toughened with PHAs [18], LLDPE [19], and SBS [20]. There are two differences between our current research and previous work [18,19,20]. The first difference is about the number of the filler’s variables. Only the effect of wood flour content has been taken into account when the previous experiments were designed (i.e., [18,19,20]). Here, the effects of both particle size and filling content of the ball-milled celluloses were considered. The second is related to modifying agent. Our previous papers reported that the wood flour/PLA composite materials toughened with different toughening agents (i.e., PHAs, SBS, and LLDPE) improved the impact strength of PLA greatly, compared with that of their untoughened counterparts [18,19,20]. However, SBS and LLDPE compromise the partial degradability of the resultant composite materials. Our current manuscript presented binary cellulose/PLA composite materials. A chemical-free facile process was used to pretreat the cellulose to access fillers. Further, there is no toughening agent added during the composite manufacture process. They are 100% bio-based PLA-based composite materials. To understand the structure–property relationships of the resultant cellulose/PLA composites, the correlations between fractal dimension and impact strength will be investigated via linear fitting method.

In this context, the objective of this study is to manufacture 100% bio-based renewable cellulose/PLA composites via a facile route and evaluate the relationships between the fractured surface morphology and impact strength of the resultant composite materials. Then, the toughening effects of the ball-milled cellulose on the matrix PLA was evaluated with impact tests. The fractal dimension of the resultant composite materials was investigated from their SEM images using the Gwyddion software with the latest version. The correlations between the fractal dimension and impact strength of the resultant cellulose/PLA composite materials were explored via linear fitting.

## 2. Materials and Method

### 2.1. Raw Materials and Cellulose Ball-Milling

Pulp cellulose fibers (southern pine, kraft bleached) from Weyerhaeuser (Seattle, WA, USA) were stored in a walk-in conditioning room at 20 °C and 65% relative humidity, with initial moisture content of 8.75 ± 0.22 wt %. PLA pellets (3052D) were supplied by NatureWorks LLC (Minnetonka, MN, USA).

Pulp cellulose fibers were ground with a planetary ball mill (PQ-N04, Across International, Livingston, NJ, USA) running at 600 rpm for different times. Two 100-mL stainless steel jars were installed. Each of them was loaded with 3.00 g of pulp cellulose fibers and 116 pieces of stainless steel balls (100 pieces of *Φ*6 and 16 pieces of *Φ*10, respectively). A laser scattering particle size analyzer (Mastersizer 3000, Malvern Instruments Ltd., Malvern, UK) was used to measure particle size and size distribution with distilled water as dispersant.

### 2.2. Composite Materials Fabrication

The ball-milled cellulose particles and PLA pellets were dried in a convection oven at 80 °C for 24 h prior to extrusion. Then, a series of mixtures of cellulose particles and PLA were blended with a twin-screw extruder (HAAKE MiniLab II, Thermo Scientific, Waltham, MA, USA) under 75 rpm and 180 °C for 5 min. The filling contents of the ball-milled cellulose were 4.8, 13.0, and 20.0 wt %, respectively. The compositions of the investigated cellulose/PLA composite materials are illustrated in Table 1.

After cooling with water, the extruded mixtures were chopped with a strand pelletizer (BT25, Bay Plastics Machinery, Bay City, MI, USA). Then, the pellets were dried in the oven at 80 °C overnight prior to injection molding (HAAKE MiniJet, Thermo Electron, Newington, NH, USA). The cylinder temperature was 190 °C. The mold temperature was 70 °C. The injection and holding pressure was set at 600 and 400 bars, respectively. The injection time was 10 s. The holding time was 30 s.

### 2.3. Impact Test

Charpy impact strength of the cellulose/PLA composites was determined with an impact tester (XJJD-5, Chengde Jinjian Testing Instrument Co. Ltd., Chengde, China), according to GB/T 16420-1996. The un-notched bars were conditioned at 50 ± 10% relative humidity and 23 ± 2 °C in a walk-in conditioning room for 48 h before testing. Five valid impact bars were tested for each composition. Their averages were presented as the results.

### 2.4. Fractured Surface Imaging

An environmental scanning electron microscope (ESEM, Quanta 200, FEI, Hillsboro, OR, USA) was used to image the impact surfaces of the resultant composites after being coated with a conducting layer of gold sputter coater. The thickness of the gold layer was about 10 nm when the spraying time was set at 5 min.

### 2.5. Fractal Analysis

The fractal dimension of the fractured surfaces was calculated with the Gwyddion software (Version 2.47, Czech Metrology Institute, Brno, Czech Republic) according to their SEM images. This free image analysis program offers four different algorithms to calculate fractal dimension: (1) Partitioning; (2) Cube counting; (3) Triangulation; and (4) Power spectrum [21]. All of the above-mentioned algorithms are modifications of the profile analysis method, in that the contours perpendicular to the plane are analyzed to determine the fractal dimension.

**Figure 1:**
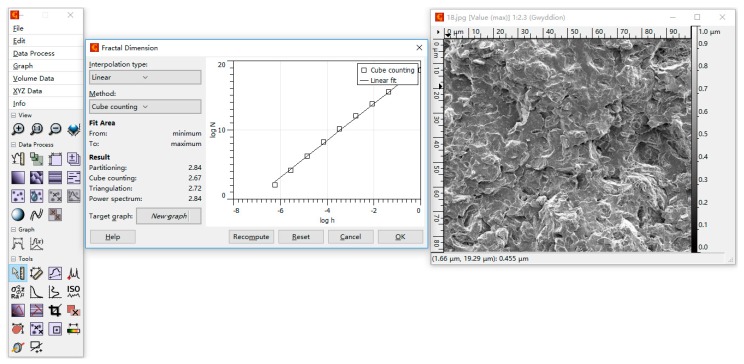
Gwyddion’s user interface to calculate fractal dimension.

The user interface of Gwyddion for calculating fractal dimension is illustrated in the middle window of Figure 1. The Cube counting method was taken as an example. The left window is the main menu of the Gwyddion software, while the right one is the figure window displaying the SEM image. For the Cube counting method, h (on the *x* axis of the middle window) means the reciprocal of lattice constant, and N (on the *y* axis) means the number of all cubes that contain at least one pixel of the image. By calculating N and h, the fractal dimension was determined by the slope of the log(N)-log(h) plot [21]. Within Gwyddion, fractal analysis was implemented in the following three steps: Data Process → Statistics → Fractal analysis [21].

## 3. Results and Discussion

### 3.1. Fractured Surface Morphology

**Figure 2:**
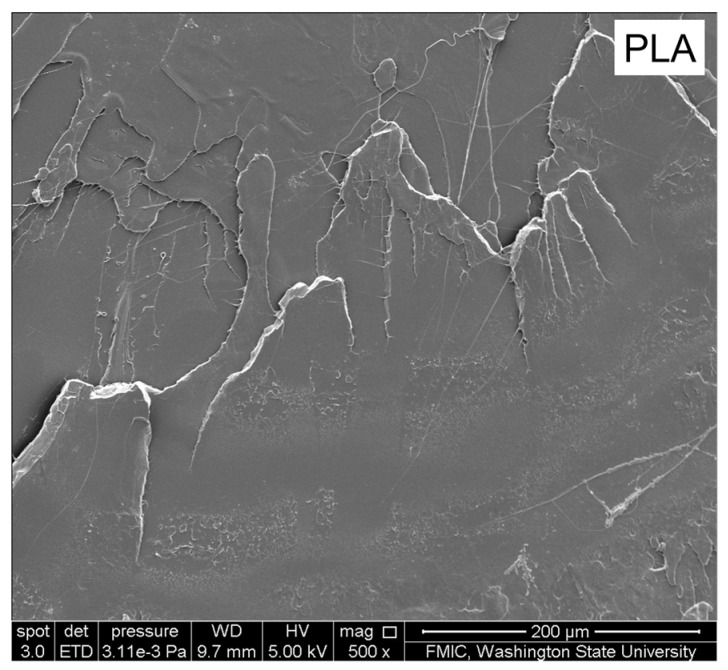
SEM images of the fracture surfaces of PLA.

The SEM image of virgin PLA exhibited a brittle fracture pattern when it experienced impact load, as shown in Figure 2. The magnification of this SEM image is 500. There were some distinct river markings on its fractured surface. The smooth, laminated surfaces confirm the inherent stiffness and brittle character of pristine PLA. The smooth and laminated surfaces with similar river markings can also be observed from its SEM image with the larger magnification. Thus, it can be predicted that they have low fractal dimension of surface morphology during the following fractal analysis.

**Figure 3:**
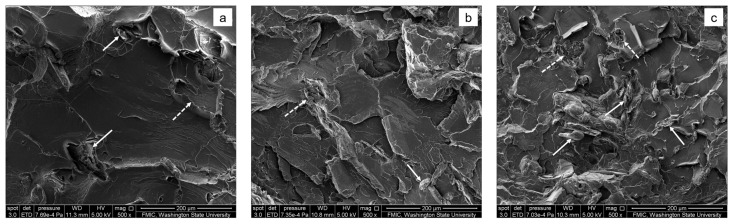

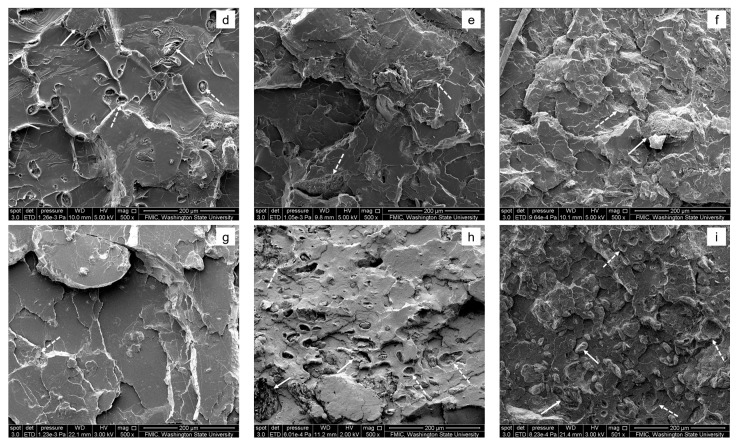
SEM images of the impact fractured surfaces of cellulose/PLA composite materials. The letters **a**–**i** are used to show their different composition for the composite materials (see the details in Table 1). The arrows mean the ball-milled cellulose particles filled in PLA. The dotted arrows mean the traces left by the cellulose particles pulled out from the matrix.

The impact fractured surfaces of the cellulose/PLA composite materials are shown in Figure 3. They were totally different from that of virgin PLA. The filled cellulose particles could be observed within the micrographs, as indicated by the arrows in Figure 3. Some of the cellulose particles were pulled out from the fractured surfaces of the PLA-based composite materials during the impact test. They could be easily recognized from the traces left by the cellulose particles pulled out, as indicated by the dotted arrows in Figure 3. There were also some micro-cracks on their fractured surfaces. This shows that the binary composite materials experienced a pull-out fracture pattern during the impact test process, similar to that of the PLA-based WPC toughened with PHAs [2]. At the same time, their fractured surfaces became rougher and more irregular with the increasing filling content (each row, from left to right, in Figure 3) and the decreasing particle size (each column, from the top down, in Figure 3) of the ball-milled celluloses. The PLA-based composites filled with large cellulose particles (i.e., 120 μm; in a, d, and g of Figure 3) had smooth and laminated fractured surfaces somewhat similar to that of PLA, while the composite materials filled with small particles (i.e., 38.9 μm; in c, f, and i of Figure 3) showed vague and rough surfaces with some ductile fracture characteristics. For a certain kind of composite materials (e.g., CaCO_3_/PLLA composite materials [17] and PLA-based WPC [18,19,20]), the rougher its impact fractured surface is, the tougher it will be. Accordingly, it will absorb more work of fracture, show higher impact resistance, and have higher fractal dimension, which will be investigated via impact test and fractal analysis.

### 3.2. Impact Strength

**Figure 4:**
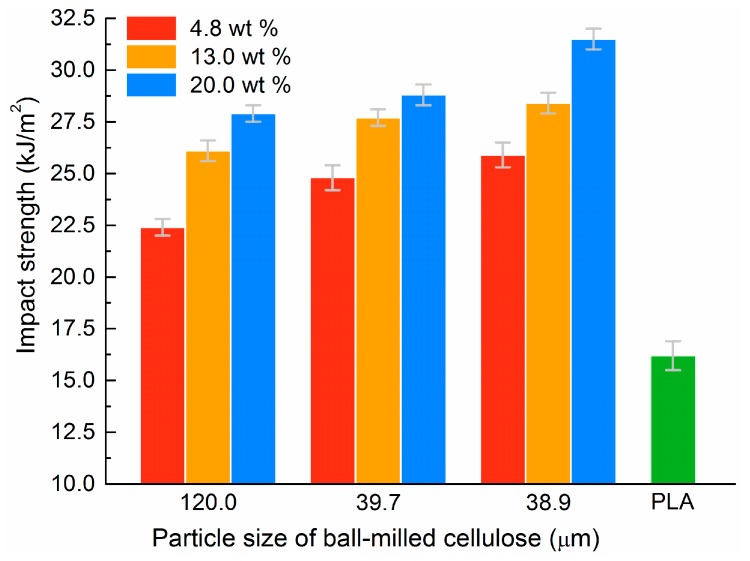
Impact strength of the cellulose/PLA composite materials. The green bar means the impact strength of pristine PLA, which is used as reference.

The impact strength of pristine PLA and the cellulose/PLA composite materials are depicted in Figure 4. The relatively low impact strength of pristine PLA (16.2 ± 0.7 kJ/m^2^) showed its inherent brittleness, which confirms the previous conclusion drawn from its smooth and laminated fracture surfaces. All of the cellulose-reinforced composite materials illustrated enhanced impact resistance compared with that of virgin PLA. For example, the PLA composite materials reinforced with the ball-milled celluloses at 4.8 wt % and large particle size (i.e., 120.0 μm) exhibited impact strength of 22.4 ± 0.4 kJ/m^2^ (138% that of pristine PLA). This shows that filling cellulose fibers into PLA can greatly improve its toughness. This is consistent with Graupner’s report [11].

Furthermore, the impact strength of the binary composite materials increased with both the increasing filling content and the decreasing particle size of the ball-milled cellulose. The maximum impact strength reached 31.5 ± 0.5 kJ/m^2^ (194% that of pristine PLA) when the ball-milled celluloses were at 20.0 wt % and small particle size (i.e., 38.9 μm). In fact, the effects of particle size and filling content of the ball-milled cellulose on their impact strength can be concluded qualitatively from the SEM images of their fractured surfaces shown in Figure 3 from upper-left to lower-right. In other words, the ductile fracture characteristics became increasingly obvious for the resultant composite materials during the impact test, due to the increasing filling content and the decreasing particle size of the ball-milled celluloses.

### 3.3. Fractal Dimension

The fractal dimensions of the impact fractured surfaces were calculated with the Gwyddion software using their individual SEM images, according to four algorithms: Cube counting, Triangulation, Variance, and Power spectrum [21]. The calculated fractal dimension of pristine PLA was 2.55, 2.63, 2.76, and 2.77, respectively. This shows that the fracture surface of PLA could be characterized in terms of a single numerical parameter (i.e., its fractal dimension). All values for PLA are between 2 and 3, meaning that the impact fractured surfaces of pristine PLA had some 3-dimensional features, although they look rather smooth under electron microscope. The Power spectrum method got the highest fractal dimension, followed by the Variance and Triangulation algorithms, while the Cube-counting method got the smallest fractal dimension for the same SEM image. The reason lies in their different algorithms within the Gwyddion software [21]. The Variance method is based on the scale dependence of the variance of fractional Brownian motion. The Power spectrum method is based on the power spectrum dependence of the variance of fractional Brownian motion. The Cube counting algorithm is derived directly from a definition of box-counting fractal dimension. The Triangulation method is very similar to the Cube-counting, and is also based directly on the box-counting fractal dimension definition.

Fractal dimensions for the fractured surfaces of the cellulose/PLA composite materials are presented in Table 2. Their individual calculated fractal dimensions are higher than that of virgin PLA, which confirms that the impact fractured surfaces for the composite materials are indeed more irregular than that of pristine PLA. This shows that the Gwyddion program can provide a reasonable parameter for the quantitative description of the irregularity of fracture surfaces for both pristine PLA and cellulose/PLA composite materials.

Liang et al. reported that the fractal dimensions of the fracture surface for PLLA composites filled with nano-sized calcium carbonate are within the range of 1.76 to 1.97 [17], which are lower than our results. One possible reason lies in the different size of fillers (nanometer in Liang’s paper vs micrometer in our work): large particles usually introduce more interfacial imperfections (e.g., micro-voids and micro-cracks), which leads to a rougher fracture surface and a higher fractal dimension. The more important reason might be attributed to the different algorithms used.

Here, the Power spectrum algorithm still got the highest fractal dimension (except for Samples g–i), followed by Variance and Triangulation algorithms for the cellulose/PLA composite materials. The Cube counting method got the smallest fractal dimension for the same SEM image. Furthermore, the calculated fractal dimension for the cellulose/PLA composite materials increased with the increasing filling content and the decreasing particle size of the ball-milled celluloses, which is consistent with the preliminary conclusion drawn by comparing their individual SEM images. To understand the microstructure–property relationships of the cellulose/PLA composite materials quantitatively, the impact strength and their individual fractured surface morphology (i.e., fractal dimension) are investigated via linear fitting method in the following section.

### 3.4. Correlation between Fractal Dimension and Impact Strength

**Figure 5:**
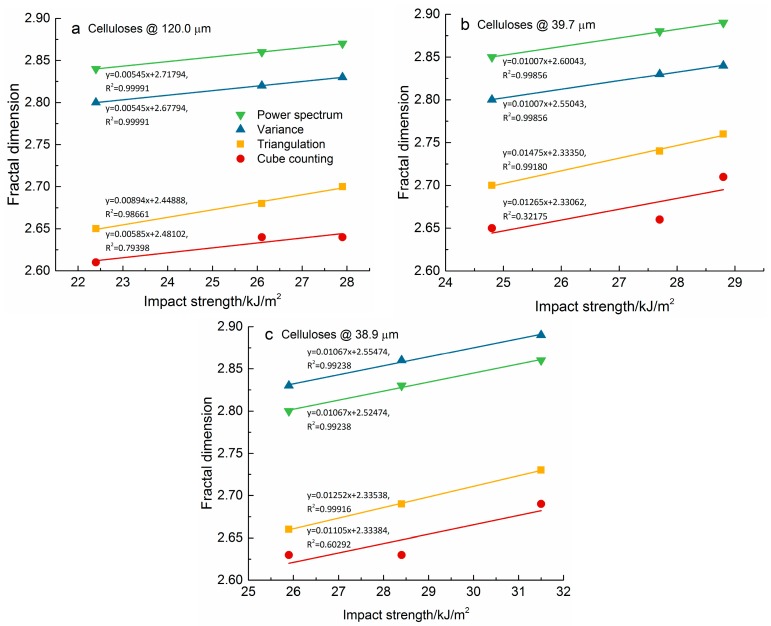
Correlation between fractal dimension and impact strength of the PLA-based composite materials reinforced with ball-milled celluloses. The particle size of ball-milled cellulose shown in (**a**–**c**) of Figure 5 is 120.0, 39.7, and 38.9 μm, respectively.

Linear fitting was used to investigate the relations between fractal dimension of the impact fractured surfaces and impact strength of the cellulose/PLA composite materials. Two indexes—Pearson’s correlation coefficient (PCC) and goodness of fit—were used to evaluate the linear fitting. PCC is the covariance of two variables X and Y divided by the product of their standard deviations, which is used to measure the linear correlation between X and Y. The value for a PCC ranges from −1 to 1, where 1 implies that there is positive linear correlation between X and Y perfectly, 0 means no linear correlation, and −1 is total negative linear correlation between them. Goodness of fit of a statistical model describes how well it fits a set of observations, which is indicated by a coefficient of determination, R-squared (R^2^). The value for R^2^ ranges from 0 to 1, where 1 indicates a perfect fit. The fitting results for the PLA-based composite materials reinforced with the ball-milled celluloses at different particle size (120.0, 39.7, and 38.9 μm) are shown in a, b, and c of Figure 5, including their individual linear equation and the adjusted R^2^.

For the PLA-based composite materials reinforced with ball-milled celluloses at different particle sizes, the fractal dimensions of the impact fractured surfaces increased with the increasing filling content for the above-mentioned algorithms (Figure 5). All of the Pearson’s correlation coefficients were greater than 0.8. The positive and high correlation coefficients imply that the linear equations can describe the relationships between fractal dimension and impact strength, with nearly all the data points lying on the lines for which impact strength increase with fractal dimension. This shows that there are positive correlations between fractal dimension of the fractured surfaces and impact strength of the cellulose/PLA composite materials. In fact, a simple proportionality relationship between the work of fracture and fractal dimension of its fracture surface is established for ceramic composites using SEM images [24]. A positive correlation was reached between the fractal dimension and resistivity of copper-tungsten films deposited on silicon wafers when power spectral density was used to calculate the fractal dimension of AFM images [26]. In addition, there was a linear correlation between impact strength and fractal dimensions of the fracture surface for PLLA composites filled with nano-sized calcium carbonate when the fractal dimension was more than 1.88 [17]. The positive correlations between the fractal dimension of their fractured surfaces and the impact strength of the cellulose/PLA composite materials are strong, due to high Pearson’s correlation coefficients.

There was some difference between the linear fitting results of fractal dimension and impact strength when the goodness of fit was taken into account. The R^2^ values related to the fractal dimensions from the Power spectrum, Variance, and Triangulation algorithms are very close to 1 (the minimum is 0.98661), as shown in Figure 5. However, the R^2^ values related to the fractal dimensions from the Cube counting algorithm are much lower than 1 (the maximum is 0.79398). In fact, the minimum R^2^ is 0.32175 for the Cube counting method. This means that the linearity between fractal dimension and impact strength are much stronger for the Power spectrum, Variance, and Triangulation methods than for the Cube counting method.

## 4. Conclusions

The PLA-based composite materials reinforced with ball-milled celluloses were fabricated via melting extrusion followed by injection molding. The un-notched impact strength was measured via impact test, and their impact fractured surfaces were imaged with SEM. Then, the fractured surfaces were investigated via fractal analysis of their SEM images. Linear fitting was used to research the correlations between fractal dimension and impact strength.

The results show that filling the ball-milled celluloses into PLA greatly improved its impact toughness. The cellulose/PLA composite materials illustrated a different fracture pattern from that of pristine PLA. The fractal dimensions of the impact fractured surfaces for the resultant composite materials increased with the increasing filling content and the decreasing particle size of the ball-milled celluloses. There were highly positive correlations between fractal dimension of the fractured surfaces and impact strength of the cellulose/PLA composites. However, the linearity between fractal dimension and impact strength was much stronger for the Power spectrum, Variance, and Triangulation method than that for the Cube counting method, due to the different R-squared values. This work will shed new light on research techniques for the structure–property relationship of polymer composites.

## Figures and Tables

**Table 1:** Compositions of the cellulose reinforced polylactide (PLA)-based composite materials.

Materials Code	Ball-Milled Cellulose		Filling Content/wt %
	Ball-Milling Time/min	Average Particle Size/μm	
Sample a	10	120.0	4.8
Sample b			13.0
Sample c			20.0
Sample d	30	39.7	4.8
Sample e			13.0
Sample f			20.0
Sample g	60	38.9	4.8
Sample h			13.0
Sample i			20.0

**Table 2:** Fractal dimensions of the impact fractured surfaces for the cellulose/PLA composite materials within the Gwyddion program using different algorithms.

Materials Code	Fractal Dimension			
	Cube Counting	Triangulation	Variance	Power Spectrum
Sample a	2.61	2.65	2.80	2.84
Sample b	2.64	2.68	2.82	2.86
Sample c	2.64	2.70	2.83	2.87
Sample d	2.65	2.70	2.80	2.85
Sample e	2.66	2.74	2.83	2.88
Sample f	2.71	2.76	2.84	2.89
Sample g	2.63	2.66	2.83	2.80
Sample h	2.63	2.69	2.86	2.83
Sample i	2.69	2.73	2.89	2.86
